# Utilization of Outpatient Eye Care Services in Taiwan: A Nationwide Population Study

**DOI:** 10.1155/2020/2641683

**Published:** 2020-01-08

**Authors:** Chia-An Hsu, Sheng-Huang Hsiao, Min-Huei Hsu, Ju-Chuan Yen

**Affiliations:** ^1^Department of Medical Education, Taipei Veterans General Hospital, Taipei, Taiwan; ^2^Department of Neurosurgery, Ren-Ai Branch, Taipei City Hospital, Taipei, Taiwan; ^3^National Chengchi University, Taipei, Taiwan; ^4^Graduate Institute of Data Science, College of Management, Taipei Medical University, Taipei, Taiwan; ^5^Department of Neurosurgery, Wan-Fang Hospital, Taipei Medical University, Taipei, Taiwan; ^6^Graduate Institute of Biomedical Informatics, College of Medical Science and Technology, Taipei Medical University, Taipei, Taiwan; ^7^Department of Ophthalmology, Ren-Ai Branch, Taipei City Hospital, Taipei, Taiwan; ^8^Department of Education and Research, Taipei City Hospital, Taipei, Taiwan

## Abstract

**Introduction:**

A study based on the Taiwanese National Health Insurance Research Database (NHIRD) to reveal the ocular diseases landscape.

**Materials and Methods:**

This study comprised all ophthalmological outpatient patient visits (*n* = 6,341,266) in the Taiwanese longitudinal NHIRD 2000. Descriptive analytics based on 15 disease categories of ICD-9-CM and 10 tiers of age categories was performed with SAS for Windows 9.3 (SAS Institute, Inc., Cary, NC, U.S.A.).

**Results:**

The average frequency of visits was 0.7 visits per year. The mean age was 36.2 years old. Bimodal peak of visits in the first, second, and eighth decade of life was revealed. Conjunctiva is the most dominant disease category throughout life while different categories play major roles in each decade of life. The most frequent disease code of each category was listed.

**Discussion:**

The bimodal peak of visits revealed the age group of the most prominent ocular disease burden. Peak in school age population can be partially explained by the nationwide vision screening program, while aging accounts for the lens disorder and glaucoma of the senile peak. The disease category frequency variation among age categories reflects the development and aging of the eye. The most frequent disease codes of each category highlight disease of importance for primary practitioners and ophthalmologists.

**Conclusion:**

Taiwanese longitudinal NHIRD was used to reveal the ophthalmological disease landscape. The epidemiological insight, while limited in clinical presentation and economic impact, enables physicians and policy makers to improve the overall vision health of the population.

## 1. Introduction

In Taiwan, the government launched the National Health Insurance (NHI) as a mandate on March 1, 1995; the entire population is virtually enrolled [1]. The National Health Insurance is a compulsory, a single-payer system with universal coverage, i.e., all citizens in Taiwan are contained in this system. The claims data for reimbursement (National Health Insurance Research Database, NHIRD) have been released by the National Health Insurance Administration for research since 1996. These claims data include payment, demographics, and disease-coding data.

There had been quite a few ophthalmology publications regarding disease associations [2, 3]. However, none attempted to construct the big picture of ophthalmology service utilization. Therefore, this study attempts to provide such information through analyzing a subdataset of NHIRD.

The Longitudinal Health Insurance Database 2000 (LHID2000) is a subdataset of the National Health Insurance Research Database (NHIRD), which includes all claims data (from 1996 to 2008) of one million beneficiaries who were randomly selected from the system in 2000. There was no significant difference in age, sex, or average-insured payroll-related premiums between the sample group and all enrollees.

By analyzing the LHID2000 dataset, this study aims to portrait the previously mentioned big picture of eye care service utilization in Taiwan. It is expected that such information could help policy makers better understand the strength and deficiencies in the current eye care service and provide crucial guidance and information to all ophthalmologists, training or practicing. Consequentially, provide better eye care service to the entire population.

## 2. Materials and Methods

This study consisted of all patient visits (*n* = 6,341,266) in the Longitudinal Health Insurance Database 2000 which included patients who visited ophthalmology outpatient services (department 10) at least once from January 1, 2000, through December 31, 2008. The data were analyzed based on ICD-9-CM codes. Demographic data, including sex and age, were recorded. The disease codes were categorized by fifteen disease categories (Table 1).

This study was approved by the institutional review board of Taipei Medical University, Taiwan. Since this study analyzed deidentified data, the review board waived the requirement for written informed consent from the patients involved.

SAS for Windows 9.3 (SAS Institute, Inc., Cary, NC, USA) was used to perform descriptive statistics analyzing demographic characteristics and ICD-9-CM disease coding.

## 3. Results

### 3.1. Demographic Data and General Information

There were 6,341,266 patient visits among the studied population (LHID2000), including patients who visited ophthalmology outpatient services at least once from Jan. 1, 2000, to December 31, 2008. Ophthalmology ranked ninth among the 48 existing departments, contributing to 4.91% of total visits.

**Figure 1:**
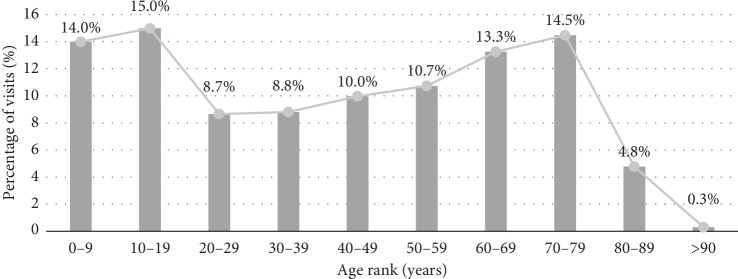
Distribution of eye care service visits among age ranks.

The mean age was 36.2 years old with a standard deviation of 24.8 years. The sex ratio (Male versus Female, M/F) was 0.764 among visits in ophthalmology department; that of the entire dataset was 0.983. The average frequency of visit was 0.7 visits per year. Grouping by age ranks revealed bimodal peaks: the former in the first (14%) and second (14.99%) decade of life; the latter in the eighth (14.88%) decade of life (Figure 1).

### 3.2. Distribution of ICD-9-CM Disease Code among Fifteen Disease Categories and the Most Frequent Disease Code in Each Category

Table 2 shows the overall distribution of ICD-9-CM disease code counts among fifteen disease categories. Conjunctival disorders ranked first, contributing 41.32% of all visits, disorders of refraction and accommodation and visual disturbances ranked second (15.57%), disorders of eyelid and orbit as third (9.01%), and disorders of lens (cataract) as fourth (8.54%), followed by disorders of cornea (4.71%), trauma (3.94%), disorders of retina (3.12), disorders of lacrimal system (2.67%), glaucoma (2.66%), and other minor contributors.

Table 3 demonstrates the most frequent ICD-9-CM disease code in each category.

### 3.3. Three Most Frequent Disease Categories among Each Decade of Life

Table 4 lists the three most frequent disease categories in each decade of life. Conjunctival disorders remained dominant throughout the entire life, contributing around forty percent of all visits. Refraction and accommodation disorders came second in the first two decades of life and tapered rapidly afterwards. Eyelid and orbit disease remained second to third throughout the first five decades of life. Trauma came third in the fourth and fifth decades of life. Lens disorders came second after the sixth decade of life. Glaucoma is the third most frequent disease category from the seventh to ninth decade of life.

## 4. Discussion

### 4.1. Bimodal Peak of Age Distribution in Ophthalmology Clinic Visits

In the studied population, patients of the first (14%), second (15%), and eighth (14.5%) decade of life contributed the most visits.

During the first and second decade of life, conjunctiva disorders predominates the clinic visits; refraction, accommodation, and visual disturbances is the second most frequent category and makes up around thirty percent of the visits. Such phenomenon is likely due to the policy of nationwide annual vision screening [4] for students of elementary, junior high, and senior high school, which refers students that failed the screening test to the ophthalmology clinic for further examination and treatment.

On the contrary, during the eighth decade of life, lens disorder and glaucoma are the two most frequently encountered disease categories. Cataract and glaucoma start to inflict the population from the sixth to seventh decade of life and is likely the most important contributor to this peak [5, 6].

Overall, the above findings suggested that ocular disease burden is most prominent in the school age and senile population.

### 4.2. Dominance of Disorders of Conjunctiva and the Variation of Frequencies of Disease Categories among Each Decade of Life

Of the fifteen disease categories, disorders of conjunctiva are the most dominant in each decade of life, making up to 34∼48% of all visits. This likely suggests the excellent accessibility of Taiwan's eye clinic since even a mild but frequent disease such as chronic conjunctivitis could receive timely care.

Moreover, the variation of the frequencies of disease categories among each decade of life reflected the epidemiological variation of ocular disease throughout life.

As for the first and second decade of life, refraction, accommodation, and visual disturbances are the most important contributors. Besides the previously mentioned nationwide screening program, it also reflects the influence of the developing eye [7]. Since the refractive development stabilizes in around the second decade of life, the preschool and school age population are also most prone to refraction error and its complication such as amblyopia [8].

Disorders of eyelid and orbit likely reflect another group of mild but frequent disease. For instance, the most frequent disease code in this category is hordeolum internum. This category of diseases played a major role in the visits of young adults and middle age population, which likely reflects the insignificant ocular disease burden in this age group.

As the eye undergoes degenerative changes, disorders of lens disorders and glaucoma start to inflict the population [9]. Disorder of lens soared to the second most frequent category in the sixth decade of life and remained significant afterwards. Glaucoma caught up as the third most frequent category in the seventh decade and remained so for the following two decades.

The variation of ocular disease epidemiology among each decade of life depicted typical ocular diseases a Taiwanese patient might encounter throughout his or her life. It highlights significant disease categories for both ophthalmologists and primary practitioners to be aware of when caring patients from each age group.

Additionally, the major disease category in the two age groups of the most prominent ocular disease burden should draw the attention of public health policy makers. Policies improving the care of refraction, accommodation, and visual disturbances in school age population and that of alleviating disease burden of cataract and glaucoma are of greatest importance.

### 4.3. Most Frequent Disease Code in Each Disease Category

The most frequent disease code in each disease category highlighted significant diseases especially for ophthalmologists and primary practitioners.

It is highly unlikely for a primary practitioner to be able to familiarize oneself of every ocular disease. However, being familiar with the most frequent disease in each category enables the practicing nonspecialist to appropriately refer patients to ophthalmologists and have a general concept of the patient's ocular comorbidities [10].

As for the training ophthalmologists, learning the landscape of ocular disease and getting familiar with the most frequent endemic disease should be a priority. On the contrary, practicing ophthalmologists should always consider the mentioned disease when diagnosing a patient.

By taking the epidemiologic insight of ocular disease into consideration, the primary practitioners gain crucial insights of ocular disease. The training ophthalmologists could speed up the process of familiarizing with the disease landscape and the practicing ones would be able to improve their quality of care by making diagnosis with greater accuracy.

## 5. Strength and Limitations of the Study

This study was conducted based on the universal, single-payer longitudinal National Health Insurance Database in Taiwan. The longitudinal flow of the ophthalmological disease landscape is probed. The enormous amount of clinic visits (*n* = 6,341,266) analyzed implicated that the landscape depicted by this study is highly representative of the entire population.

However, cost was not taken into consideration in this study. Thus, the economic impact of each disease cannot be properly estimated in this study. Also, since the chart review was not conducted, no clinical presentations or chief complaints could be reviewed in this study.

Future study, including chart review and retrieving financial data of NHIRD, might complement the deficiency of this study. Nevertheless, the importance of the epidemiological insight revealed by this study is crucial to every primary practitioner and ophthalmologist in Taiwan.

## 6. Conclusions

The Taiwanese National Health Insurance Database was used to examine the longitudinal flow of ophthalmological presentations based on 15 ICD-9-CM disease categories and ten age categories. The results revealed bimodal peaks of visits in the school age and senile population. Variation of frequencies of each disease categories revealed a typical ocular disease pattern in Taiwanese patients, and the most frequent disease code in each disease category highlights significant and frequent diseases in Taiwan.

While this study is limited in clinical presentation and economic impact, the epidemiological insight is critical for primary practitioners, ophthalmologists, and policy makers. By learning the big picture of Taiwan's eye care utilization, better care could be delivered and more optimal public health policies could be made. Ultimately, promoting the vision health of the population of Taiwan.

## Figures and Tables

**Table 1:** Disease categorization according to ICD-9-CM codes.

Disease categories	Disease code
Disorders of globe	360
Disorders of retina	361, 362
Disorders of uvea (choroid, iris, ciliary body)	363, 364
Glaucoma	365
Disorder of lens (cataract)	366
Disorders of refraction and accommodation and visual disturbances	367, 368
Blindness and low vision	369
Disorders of cornea	370, 371, 918
Disorders of conjunctiva	372
Disorders of eyelid and orbit	373, 374, 376
Disorders of lacrimal system	375
Disorders of optic nerve and visual pathway	377
Strabismus and other disorders of binocular eye movements	378
Others, unclassified including vitreous disorders	379
Trauma	870, 921, 930, 940, 950

**Table 2:** Distribution of ICD-9-CM codes according to disease categories.

Disease categories	Percentage (%)
Disorders of conjunctiva	41.32
Disorders of refraction and accommodation	15.57
Disorders of eyelids and orbits	9.01
Disorders of lens	8.54
Disorders of cornea	4.71
Trauma	3.94
Disorders of retina	3.12
Disorders of lacrimal system	2.67
Glaucoma	2.66
Others, unclassified including vitreous disorders	1.7
Disorders of uvea (choroid, iris, ciliary body)	0.43
Disorders of globe	0.29
Strabismus and other disorders of binocular eye movements	0.18
Disorders of optic nerve and visual pathway	0.12
Blindness and low vision	0.04
Total percentage	94.3

**Table 3:** Most frequent ICD-9-CM disease code in each category.

Disease categories	Most frequent disease code (percentage of entire population)
Disorders of globe	Progressive high myopia, 36021 (0.07%)
Disorders of retina	Proliferative diabetic retinopathy, 36202 (0.51%)
Disorders of uvea (choroid, iris, ciliary body)	Acute anterior uveitis, 36400 (0.22%)
Glaucoma	Primary open angle glaucoma, 36511 (0.62%)
Disorder of lens (cataract)	Nuclear sclerosis, 36610 (5.34%)
Disorders of refraction and accommodation and visual disturbances	Myopia, 3671 (6.1%)
Blindness and low vision	Unspecified visual loss, 3699 (0.04%)
Disorders of cornea	Punctate keratitis, 37021 (1.2%)
Disorders of conjunctiva	Chronic conjunctivitis, 37210 (11.21%)
Disorders of eyelid and orbit	Hordeolum internum, 37312 (2.47%)
Disorders of lacrimal system	Tear film insufficiency, 37515 (2.0%)
Disorders of optic nerve and visual pathway	Optic atrophy, 37710 (2.0%)
Strabismus and other disorders of binocular eye movements	Exotropia, 37810 (0.06%)
Others, unclassified including vitreous disorders	Vitreous opacity, 37924 (0.51%)
Trauma	Corneal foreign body, 9300 (1.89%)

**Table 4:** Three most frequent disease categories in each decade of life.

Age (years)	1st	2nd	3rd
0–9	Conjunctiva (47.0%)	Refraction (30.1%)	Eyelid/orbit (6.4%)
10–19	Conjunctiva (39.1%)	Refraction (36.1%)	Eyelid/orbit (7.0%)
20–29	Conjunctiva (48.6%)	Eyelid/orbit (10.9%)	Cornea (6.2%)
30–39	Conjunctiva (43.7%)	Eyelid/orbit (10.3%)	Trauma (6.5%)
40–49	Conjunctiva (48.2%)	Eyelid/orbit (7.5%)	Trauma (6.8%)
50–59	Conjunctiva (44.9%)	Lens (6.0%)	Eyelid/orbit (5.5%)
60–69	Conjunctiva (38.9%)	Lens (20.6%)	Glaucoma (6.0%)
70–79	Conjunctiva (34.2%)	Lens (26.9%)	Glaucoma (7.2%)
80–89	Conjunctiva (33.8%)	Lens (23.7%)	Glaucoma (8.6%)
>90	Conjunctiva (38.0%)	Lens (9.4%)	Eyelid/orbit (9.4%)

## Data Availability

The data used to support the findings of this study are available from the corresponding author upon request.
